# The Effect of a Transdermal Scopolamine Patch on Postoperative Nausea and Vomiting after Retromastoid Craniectomy with Microvascular Decompression: A Preliminary Single Center, Double-Blind, Randomized Controlled Trial

**DOI:** 10.3390/jcm9010156

**Published:** 2020-01-07

**Authors:** Hyun Hee Lee, Hyun-Mi Kim, Ji Eun Lee, Young-Tae Jeon, Sanghon Park, Kihwan Hwang, Jung Ho Han

**Affiliations:** 1 Department of Nursing, Seoul National University Bundang Hospital, Gyeonggi-do 13620, Korea; hyunhie830@snubh.org (H.H.L.); 10984@snubh.org (H.-M.K.); ppuppu20@snubh.org (J.E.L.); 2 Department of Anesthesiology, Seoul National University College of Medicine, Seoul National University Bundang Hospital, Gyeonggi-do 13620, Korea; ytjeon@snubh.org; 3 Department of Anesthesiology, Sheikh Khalifa Specialty Hospital, Ras al-Khaimah, UAE; psh1399@gmail.com; 4 Department of Neurosurgery, Seoul National University Bundang Hospital, Gyeonggi-do 13620, Korea; coolghh@gmail.com; 5 Department of Neurosurgery, Seoul National University College of Medicine, Seoul 03080, Korea

**Keywords:** microvascular decompression, postoperative nausea and vomiting, scopolamine, hemifacial spasm, trigeminal neuralgia

## Abstract

**Background:** We performed this prospective double-blind randomized controlled trial to identify the effect of a preoperative prophylactic transdermal scopolamine (TDS) patch on postoperative nausea and vomiting (PONV) after retromastoid craniectomy with microvascular decompression (RMC-MVD). **Methods:** We recruited 38 patients undergoing RMC-MVD and randomized them into two groups: the TDS group (n = 19, application of the TDS patch) and placebo group (n = 19, application of a sham patch). Nausea (as a self-reported 100-mm visual analog scale (VAS) score; range, 0 (no nausea) to 10 (worst nausea)), vomiting, and the use of antiemetics were the primary endpoints. **Results:** There was no significant difference in terms of the incidence of PONV (73.7% in the TDS group and 78.9% in the placebo group; p = 1.00) between the groups. However, the mean nausea VAS score was significantly different at arrival to the general ward (0.93 ± 1.71 in the TDS group vs. 2.52 ± 2.85 in the placebo group; p = 0.046), and throughout the study period (0.03 ± 0.07 in the TDS group vs. 0.44 ± 0.71 in the placebo group; p = 0.029). Rescue antiemetics were more frequently used in the placebo group than in the TDS group (9 (47.4%) vs. 2 (10.5%), respectively; p = 0.029). The mean number of antiemetics used throughout the study period was significantly higher in the placebo group than in the TDS group (1.37 ± 2.19 vs. 0.16 ± 0.50, respectively; p = 0.029). **Conclusions:** The preoperative prophylactic use of a TDS patch was safe and effective in the management of PONV after RMC-MVD in terms of the severity of PONV and the use of rescue antiemetics.

## 1. Introduction

Postoperative nausea and vomiting (PONV) can be defined as the presence of nausea and vomiting during the first 24 hours after a surgical procedure [1]. Despite great advances in modern neurosurgical techniques and anesthesia, PONV remains one of the most common postoperative concerns and challenges for subjects and clinicians after craniotomy. Its incidence has been reported to be high as 73% after craniotomy, and it usually leads to patient discomfort, delayed discharge from the hospital, and increased medical costs [2,3]. In addition, PONV may result in an increased risk of aspiration, intracranial hypertension, and neurological deterioration in neurosurgical patients [4]. The etiology of PONV after craniotomy may be multifactorial, and risk factors of PONV generally include a history of motion sickness, history of PONV, female gender, nonsmoking status, use of postoperative opioids, and type of surgery [3,5].

Though the type of surgery as a risk factor for PONV is still debated [6] recently, retromastoid craniectomy with microvascular decompression (RMC-MVD) has been suggested as an independent risk factor for PONV after craniotomy [7]. RMC-MVD is an effective and safe surgical procedure for hyperactive dysfunctional cranial nerve syndromes such as hemifacial spasm (HFS) and trigeminal neuralgia (TN), which are usually caused by vascular compression [8,9]. To completely alleviate vascular compression, the involved cranial nerves should be dissected along the whole intracranial portion, including the root exit/entry zone (REZ) of each cranial nerve on the brainstem surface. Because these neural structures are located close to the vestibular nerve and nucleus of the brainstem, it may be a natural phenomenon for RMC-MVD to be significantly associated with PONV [10]. However, these features related to surgical procedures suggest that PONV after RMC-MVD might have a different pathophysiology to other surgical procedures, including strabismus surgery, gynecological surgery, middle ear surgery, and breast surgery.

Nonetheless, little information exists about the proper management of PONV after RMC-MVD. Thus, approximately two-thirds of patients experience PONV after RMC-MVD, despite the use of intraoperative prophylactic medication [10]. Interestingly, application of a prophylactic transdermal scopolamine (TDS) patch showed possible protective effects against PONV in patients treated with RMC-MVD in one retrospective study, decreasing PONV incidence by 60% [10]. Scopolamine might be a drug of choice for PONV after RMC-MVD, considering the surgical features of RMC-MVD mentioned above, because scopolamine primarily acts on the central nervous system (CNS) by blocking cholinergic transmission from vestibular nuclei to higher CNS centers and from the reticular formation to the vomiting center [11]. 

Therefore, we performed this prospective double-blind randomized controlled study to investigate whether preoperative TDS patch application can have a positive effect on PONV and the use of postoperative antiemetics after RMC-MVD. 

## 2. Materials and Methods

### 2.1. Patients

The research protocol was approved by our institutional review board and registered in ClinicalTrials.gov (NCT02968082). After obtaining written and informed consent, patients scheduled for RMC-MVD (American Society of Anesthesiologists [ASA] physical status I or II, aged 18–65 years, normal liver, and kidney function) were eligible to participate unless the following exclusion criteria applied: known allergy to the study drug (scopolamine), additional emergency surgery after RMC-MVD, history of narrow-angle glaucoma, gastrointestinal obstruction, urinary tract obstruction, pregnancy, breastfeeding, or those who could not understand the visual analog scale (VAS).

After informed consent, patients were randomized into two study groups. The TDS group received a scopolamine-1.5 mg-containing patch (Kimite™; Myungmoon Pharm Co., Ltd.; Gyeonggi-do, South Korea), and the placebo group received an identical placebo patch without any drug before surgery. The patches for the two groups were identical in appearance with no identifying information. We used sequentially numbered, opaque, sealed envelopes containing the assignments; quick response (QR) code cards were computer-generated, shuffled, and then randomly inserted into the envelopes for randomization into the TDS group and a placebo group. Again, we used a simple computer-generated randomization scheme. We designed a double-blind study, which meant that surgeons, patients, and patient interviewers were blinded toward patient exposure. The assigned patch was placed on a hairless patch of skin in the contralateral mastoid area on the scheduled RMC-MVD side by an independent examiner at approximately 9 PM the night before surgery.

Subjects that experienced nausea and/or vomiting after surgery received antiemetics, including metoclopramide 10 mg IV, ondansetron 4 mg IV, and/or ramosetron 0.3 mg IV, based on a physician’s decision, considering the severity of PONV and/or a preset clinical pathway for RMC-MVD. Rescue antiemetics were given to patients whose nausea VAS was greater than 4 and who experienced more than one episode of vomiting or wanted to be treated.

### 2.2. Anesthesia

Anesthesia and monitoring were standardized for all patients as described elsewhere [3]. Briefly, patients received no preanesthetic medication. Induction of anesthesia consisted of propofol (4 µg/mL) and remifentanil (3–4 ng/mL) using target-controlled infusion. Neuromuscular blockade was performed using intravenous rocuronium 0.6 mg/kg to facilitate tracheal intubation. Propofol (2–4 µg/mL) and remifentanil (2–4 ng/mL) in oxygen and medical air (FiO_2_ 0.5) were used during the maintenance of anesthesia. Ventilation was mechanically controlled to achieve end-tidal CO_2_ between 30 and 35 mmHg. For intraoperative neurophysiology monitoring, no additional neuromuscular blocker was administered during surgery. Y.-T.J. was the unblinded personnel in the study anesthesia regimen, and no other antiemetic prophylactic drug or steroid were used during surgery. 

### 2.3. Surgery of RMC-MVD

One neurosurgeon (J.H.H.) performed all RMC-MVDs, which was described elsewhere [12]. Briefly, with the patient in the supine position, the head was rotated approximately 20 to 30° away from the affected side without the use of head-fixation. A 4–5 cm curvilinear skin incision was made along the hairline, in which three quarters were below the mastoid notch in the cases of HFS and a half were above the mastoid notch in the cases of TN. Following the identification of the digastric groove, a 2 to 2.5 cm craniectomy was performed below the digastric groove for HFS and above the digastric groove for TN. An incision of the dura mater was made along the inferoposterior margin of the sigmoid sinus. The offending vessel(s) were decompressed from the cranial nerves, after exploration of the whole intracranial portion of each involved cranial nerve, including the REZ and cisternal segment. A watertight dural closure was subsequently performed. Finally, the deep and superficial muscles and skin were approximated.

### 2.4. Outcome Measurements and Statistical Analyses

After surgery, subjects were transferred to the post-anesthesia care unit (PACU), and then to general wards usually after a one-hour recovery from anesthesia. Nausea (as a self-reported 100-mm VAS; range, 0 (no nausea) to 10 (worst nausea)), vomiting, and the use of antiemetics were the primary endpoints and were recorded upon arrival to the PACU, on transfer to the general ward, and then, at 4 h intervals until 48 h after arrival to the general ward. After the last evaluation of PONV, the TDS patch was removed. Nausea was defined as a subjectively unpleasant sensation associated with the awareness of the urge to vomit, and vomiting was defined as the forceful expulsion of gastric contents from the mouth [3]. The incidence of PONV was calculated as the sum of the number of patients with a nausea VAS score greater than 0 (except patients only with a nausea VAS scale of 0) and/or the number of patients vomiting per group.

The incidence of PONV was 69%–73% after RMC-MVD [7,10]. A retrospective study reported that only one patient among 21 patients who received an ondansetron plus TDS patch experienced nausea after RMC-MVD [10]. Considering the synergistic effect of ondansetron and the TDS patch, a reduction of 40% in PONV with a TDS patch was considered clinically significant because ondansetron can independently reduce PONV by approximately 25% [13]. The analysis showed that 27 patients in each group would be sufficient to observe the effect of a TDS patch on PONV with a level of significance of α = 0.05 and 80% power. We chose 30 patients per group, assuming a 10% drop-out rate.

We regarded a 2-sided *p*-value of 0.05 as statistically significant. Continuous variables were analyzed by Student’s *t*-test, and categorical variables were compared using the chi-square test or Fisher’s exact test. Data analyses were performed using software (IBM SPSS Statistics, Version 22 for Windows; IBM, Chicago, IL, USA).

## 3. Results

**Figure 1:**
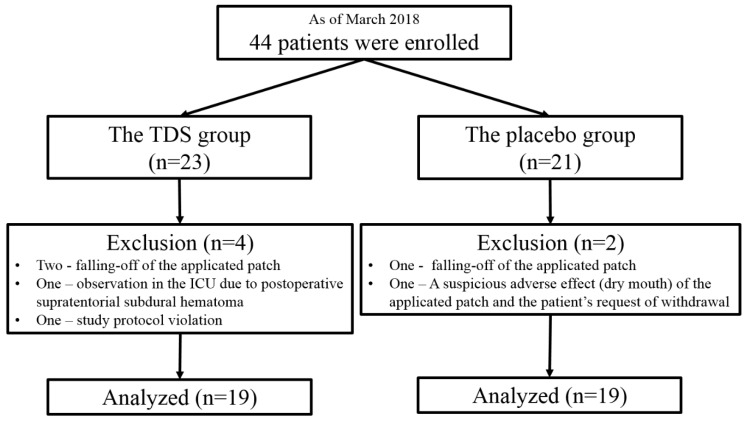
Flow diagram of the patients.

A total of 44 patients were enrolled between October 2016 and March 2018, and six were excluded during allocation or the follow-up period. Ultimately, a total of 38 patients (19 in each group) completed the study (Figure 1). In March 2018, the present study was closed due to the expiration date of the TDS patches, which were concealed and enveloped before the start of this study. Patient characteristics and information on the drugs used for postoperative pain control are summarized in Table 1.

### 3.1. The Incidence and Severity of PONV

**Figure 2:**
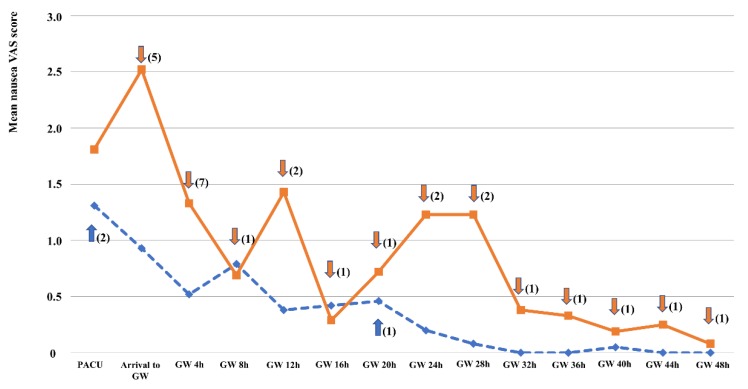
Nausea visual analog scale scores of the two groups throughout the study period (line, the placebo group; dashed line, the TDS group). Arrows with numbers indicate the number of rescue antiemetics used (upper arrow, TDS group; down arrow, placebo group). The numbers indicate the total number of rescue antiemetics used at each time (“PACU”, use of rescue antiemetics in the PACU; “Arrival to GW”, immediately after arrival to general wards; “GW 4H”, the interval right before GW 4H). No arrow means “no use of rescue antiemetics”.

There was no significant difference in the incidence of PONV (73.7% in the TDS group and 78.9% in the placebo group; *p* = 1.00). However, in terms of the severity of PONV, the mean nausea VAS scale was significantly different at arrival to the general ward (0.93 ± 1.71 in the TDS group vs. 2.52 ± 2.85 in the placebo group; *p* = 0.046). Generally, PONV was most severe at an immediate postoperative period (PACU and arrival to the general ward) and then gradually subsided until 48 hours after surgery (Figure 2). The mean nausea VAS scale was also different between the TDS group and placebo group at 24 hours, 28 hours, and 32 hours after arrival to the general ward (0.20 ± 0.39 vs. 1.23 ± 2.18, 0.08 ± 0.25 vs. 1.23 ± 2.32, and 0.00 ± 0.00 vs. 0.38 ± 0.76, respectively). However, the differences did not reach statistical significance (Table 2). The mean sum of the nausea VAS scores throughout the study period was significantly lower in the TDS group (0.03 ± 0.07 vs. 0.44 ± 0.71 in the placebo group; *p* = 0.029). However, there was no significant difference in terms of vomiting between the groups throughout the study period.

### 3.2. The Use of Rescue Antiemetics and Side Effects

Rescue antiemetics were more frequently used in the placebo group than in the TDS group (9 (47.4%) vs. 2 (10.5%) times, respectively; *p* = 0.029). The number needed to treat (NNT) was 2.7. In addition, the mean number of antiemetics used throughout the study period was significantly higher in the placebo group than in the TDS group (1.37 ± 2.19 vs. 0.16 ± 0.50, respectively; *p* = 0.029) (Table 3). The difference in the mean number of antiemetics used was distinct at the immediate postoperative period from the PACU to 4 hours after arrival to the general ward (1 (5.3%) and 0.11 ± 0.46 in the TDS group vs. 8 (42.1%) and 0.63 ± 0.83 in the placebo group; *p* = 0.019 and *p* = 0.022, respectively) (Table 3).

No patients experienced side effects related to the TDS patch in the TDS group. However, one patient, who was later identified as being assigned to the placebo group, complained of dry mouth after application of a patch and withdrew from the study.

## 4. Discussion

RMC-MVD is a surgical procedure to solve hyperactive dysfunctional cranial nerve syndromes by the manipulation of vessels close to the vomiting center of the brainstem. Specifically, the flocculus and/or vestibulocochlear nerve of the nervous system with vestibular function or balance should be directly manipulated for surgeons to clearly identify the REZ during RMC-MVD for HFS. Therefore, a high rate of PONV after RMC-MVD seems to be an inevitable result. This was clearly observed in the present study, as over 70% of patients complained of some degree of PONV in both the experimental and control groups. The difference in the incidence of PONV was not significant between the two groups, which suggests that the TDS application did not work. However, this finding depends on the definition of “the incidence of PONV” used in the present study or the “total response” being defined as no nausea, no retching, no vomiting, or no need for rescue antiemetics. This finding also demonstrates that many patients experience some degree of PONV after RMC-MVD, even with TDS prophylaxis. 

The effect of TDS prophylaxis on PONV was obvious in terms of the use of rescue antiemetics. Only two (10.5%) TDS patients needed rescue antiemetics, even though over 70% of patients experienced PONV in the TDS group. This seemed to be explained by the fact that the severity of PONV, expressed as the mean sum of the nausea VAS scores throughout the study period, was significantly lower in the TDS group. Although the difference in the nausea VAS score at each measurement interval was significant only in the immediate postoperative period (arrival to the general ward from PACU) in this study, the abundant use of rescue antiemetics in the placebo group seems to have masked the possible difference between the groups during the remaining study period after the immediate postoperative period. Such use of rescue antiemetics also seemed to be effective in reducing the severity of PONV. However, frequent use of rescue antiemetics may not only increase the overall medical cost, but also expose patients to serious complications related to antiemetics such as dyskinesia, QT prolongation, or torsade de pointes [6]. 

Adverse events of the TDS patch are not uncommon. However, most are not deemed to be serious. Dry mouth is the most frequent adverse event associated with TDS. However, there were no patients who complained about dry mouth or other adverse events in the TDS group in this study. Nonetheless, dry mouth might have been underestimated because it may not be easily differentiated from thirst caused by perioperative overnight fasting. The placed TDS or placebo patches did have a problem with falling off. During preparation for surgery and irrigation of the surgical field in the supine lateral position used for RMC-MVD, preparation and irrigation fluid ran to the contralateral side and made the patches fall off. Therefore, additional adhesive plaster may be helpful for patients who undergo surgical procedures in the head and neck area.

The “big little problem”, a term described for PONV in 1991 by Kapur [14], shows how physicians think about PONV. Although postoperative patients have ranked PONV as the most undesirable experience of all anesthesia outcomes after surgery [15], PONV has been regarded as a problem that will be solved with time. In fact, PONV gradually subsides usually within 24–48h after surgery, as was seen in the present study. However, numerous complaints from patients with PONV indicate that it is a significant and the main burden for both patients and medical personnel, especially in the early postoperative period. Thus, a protocol should be prepared to assess the actual risk of PONV in each individual patient and to prevent PONV in every kind of surgical procedure. Such a protocol for PONV prophylaxis should be cost-effective. Therefore, older, less expensive drugs such as a TDS patch would support patient groups, even those with a lower risk for PONV. 

The present study had several limitations. First, the estimated minimal sample size needed to detect differences in the primary outcome was not achieved because of the expiration of the TDS patches. However, the inadequate sample size might not affect the overall results about the incidence and severity of nausea in the present study, but the decreased sample size might mask the possible difference in terms of vomiting. Second, the present study was performed in a single center. Thus, the overall outcomes might be affected by the surgeon’s experience in RMC-MVD. However, a single surgeon (J.H.H.) performed all of the surgical procedures to maintain a uniform application of surgical stimulus and to show more clearly the prophylactic effect of TDS for PONV. Third, after randomization, the mean age of the TDS group was younger than that of the placebo group. However, an age under 50 years is generally regarded as a significant risk factor for PONV [6]. Thus, this age difference may not be a confounding factor but may be a factor masking a greater difference between the two groups than that shown in this study. Lastly, RMC-MVD was thought to be a type of surgery with a high-risk of PONV. Nonetheless, a single drug in a TDS patch was used. A combination of prophylactic drugs might have been used if there had been more information about PONV after RMC-MVD. Until now, few retrospective studies had dealt with this issue [7,10]. Thus, little evidence exists for multidrug combinations for PONV prophylaxis, and this issue should be resolved in the near future based on the results of the present study.

## 5. Conclusions

The preoperative prophylactic use of a TDS patch might be safe in the management of PONV after RMC-MVD. In particular, the TDS patch may reduce the severity of PONV and decrease the rate of rescue antiemetic use after RMC-MVD.

## Figures and Tables

**Table 1:** Characteristics of patients.

		TDS Group (*n* = 19) N (%) / Mean ± SD	Placebo Group (*n* = 19) N (%) / Mean ± SD	*p* Value
**Sex**	Male	9 (47.4)	3 (15.8)	0.079 ^§^
	Female	10 (52.6)	16 (84.2)	
**Age (year)**		46.63 ± 11.02	53.63 ± 8.780	0.037
HDCN syndrome *	HFS ^¶^	16 (84.2)	16 (84.2)	1.00 ^§^
	TN ^¶¶^	3 (15.8)	3 (15.8)	
**Symptom duration (mo)**		38.26 ± 32.62	52.66 ± 37.68	0.216 ^§^
**History of previous surgery**	Yes	13 (68.4)	12 (63.2)	1.00 ^§^
	No	6 (31.6)	7 (36.8)	
**History of PONV †**	Yes	3 (15.8)	0	0.220 ^§^
	No	10 (52.6)	12 (63.2)	
**Smoking**	Yes	3 (15.8)	2 (10.5)	1.00 ^§^
	No	16 (84.2)	17 (89.5)	
**History of Motion sickness**	Yes	9 (47.4)	7 (36.8)	0.743 ^§^
	No	10 (52.6)	12 (63.2)	
**GI disturbance**	Yes	4 (21.1)	3 (15.8)	1.00 ^§^
	No	15 (78.9)	16 (84.2)	
**ASA ‡ physical status**	I	14 (73.7)	12 (63.2)	0.728 ^§^
	II	5 (26.3)	7 (36.8)	
**Operation time (min)**		90.37 ± 23.52	83.16 ± 14.83	0.266
**Anesthetic agents**	Propofol (mL)	87.06 ± 23.85	74.72 ± 18.11	0.093
	Fentanyl (µg)	1214.71 ± 441.50	1133.33 ± 280.76	0.517
**Postoperative opioids**	Yes	10 (52.6)	16 (84.2)	0.079 ^§^
	No	9 (47.4)	3 (15.8)	
**Mean Opioid MME**		2.75 ± 0.79	2.66 ± 0.63	0.740

* HDCN syndrome, hyperactive dysfunctional cranial nerve syndrome; † PONV, postoperative nausea and vomiting; ‡ ASA, American Society of Anesthesiologists; ^¶^ HFS, hemifacial spasm; ^¶¶^ TN, trigeminal neuralgia; TDS, transdermal scopolamine; MME, morphine milligram equivalent; ^§^ Fisher’s Exact test. Bolded values indicate *p* < 0.05.

**Table 2:** Nausea visual analogue scale (VAS) score and vomiting throughout the study period.

		TDS Group (*n* = 19) Mean ± SD	Placebo Group (*n* = 19) Mean ± SD	*p* Value
PACU*	Nausea VAS	1.31 ± 2.22	1.81 ± 2.16	0.487
	Vomiting	0	0.05 ± 0.23	0.331
**Arrival to general wards**	Nausea VAS	0.93 ± 1.71	2.52 ± 2.85	0.046
	Vomiting	0	0.05 ± 0.23	0.331
**GW 4 h†**	Nausea VAS	0.52 ± 1.00	1.33 ± 2.12	0.114
	Vomiting	0	0.11 ± 0.32	0.163
**GW 8 h**	Nausea VAS	0.79 ± 1.39	0.69 ± 1.16	0.821
	Vomiting	0.05 ± 0.23	0.16 ± 0.37	0.305
**GW 12 h**	Nausea VAS	0.38 ± 0.81	1.43 ± 3.02	0.162
	Vomiting	0	0.11 ± 0.46	0.331
**GW 16 h**	Nausea VAS	0.42 ± 0.89	0.29 ± 0.52	0.596
	Vomiting	0	0	-
**GW 20 h**	Nausea VAS	0.46 ± 1.18	0.72 ± 1.70	0.590
	Vomiting	0.05 + 0.23	0	0.331
**GW 24 h**	Nausea VAS	0.20 ± 0.39	1.23 ± 2.18	0.057
	Vomiting	0	0.05 ± 0.23	0.331
**GW 28 h**	Nausea VAS	0.08 ± 0.25	1.23 ± 2.32	0.051
	Vomiting	0	0	-
**GW 32 h**	Nausea VAS	0	0.38 ± 0.76	0.050
	Vomiting	0	0	-
**GW 36 h**	Nausea VAS	0	0.33 ± 0.97	0.163
	Vomiting	0	0.06 ± 0.24	0.331
**GW 40 h**	Nausea VAS	0.05 ± 0.15	0.19 ± 0.52	0.261
	Vomiting	0	0	-
**GW 44 h**	Nausea VAS	0	0.25 ± 0.58	0.104
	Vomiting	0	0	-
**GW 48 h**	Nausea VAS	0	0.08 ± 0.19	0.165
	Vomiting	0	0	-

* PACU, post anesthesia car e unit; † GW 4 h, four hours after arrival to general wards. Bolded values indicate *p*< 0.05.

**Table 3:** Use of rescue antiemetics.

			TDS Group (*n* = 19) N (%) / Mean ± SD	Placebo Group (*n* = 19) N (%) / Mean ± SD	*p* Value
Within GW 4 h*	Use of rescue antiemetics	Yes	1 (5.3)	8 (42.1)	0.019
		No	18 (94.7)	11 (57.9)	
	Total No. of use of rescue antiemetics		0.11 ± 0.46	0.63 ± 0.83	0.022
**Within GW 24 h**	Use of rescue antiemetics	Yes	2 (10.5)	9 (47.4)	0.029
		No	17 (89.5)	10 (52.6)	
	Total No. of use of rescue antiemetics		0.16 ± 0.50	1.00 ± 1.49	0.029
**Within GW 48 h**	Use of rescue antiemetics	Yes	2 (10.5)	9 (47.4)	0.029
		No	17 (89.5)	10 (52.6)	
	Total No. of use of rescue antiemetics		0.16 ± 0.50	1.37 ± 2.19	0.029

*“Within GW 4 h” means the interval from “post anesthesia care unit” to “4 h after arrival to general wards”.
